# Transcriptomic Diversity in the Livers of South African Sardines Participating in the Annual Sardine Run

**DOI:** 10.3390/genes12030368

**Published:** 2021-03-04

**Authors:** Arsalan Emami-Khoyi, Rynhardt Le Roux, Matthew G. Adair, Daniela M. Monsanto, Devon C. Main, Shilpa P. Parbhu, Claudia M. Schnelle, Carl D. van der Lingen, Bettine Jansen van Vuuren, Peter R. Teske

**Affiliations:** 1 Centre for Ecological Genomics and Wildlife Conservation, Department of Zoology, University of Johannesburg, Auckland Park 2006, South Africa; ekarsalan@gmail.com (A.E.-K.); rynhardt07@gmail.com (R.L.R.); mattgadair@gmail.com (M.G.A.); dmonsanto119@gmail.com (D.M.M.); maindevon@gmail.com (D.C.M.); shilpa.parbhu@yahoo.com (S.P.P.); cmschnelle@gmail.com (C.M.S.); bettinevv@uj.ac.za (B.J.v.V.); 2 Branch: Fisheries Management, Department of Environment, Forestry and Fisheries, Private Bag X2, Vlaeberg 8012, South Africa; carl.vanderlingen@gmail.com; 3 Department of Biological Sciences and Marine Research Institute, University of Cape Town, Private Bag X3, Rondebosch 7700, South Africa

**Keywords:** *Sardinops sagax*, sardine run, liver transcriptome, migration, RNA-seq, climate change

## Abstract

During austral winter, the southern and eastern coastlines of South Africa witness one of the largest animal migrations on the planet, the KwaZulu-Natal sardine run. Hundreds of millions of temperate sardines, *Sardinops sagax*, form large shoals that migrate north-east towards the subtropical Indian Ocean. Recent studies have highlighted the role that genetic and environmental factors play in sardine run formation. In the present study, we used massively parallel sequencing to assemble and annotate the first reference transcriptome from the liver cells of South African sardines, and to investigate the functional content and transcriptomic diversity. A total of 1,310,530 transcripts with an N50 of 1578 bp were assembled de novo. Several genes and core biochemical pathways that modulate energy production, energy storage, digestion, secretory processes, immune responses, signaling, regulatory processes, and detoxification were identified. The functional content of the liver transcriptome from six individuals that participated in the 2019 sardine run demonstrated heterogeneous levels of variation. Data presented in the current study provide new insights into the complex function of the liver transcriptome in South African sardines.

## 1. Introduction

The South African coastline is unique in that it is strongly influenced by two of the Earth’s major ocean currents, the cold Benguela Current and the warm Agulhas Current. This distinctive dynamic support some of the richest marine biodiversity on the planet [1]. During austral winter, typically between May and early June, the coastlines of the Eastern Cape (EC) and KwaZulu-Natal (KZN) provinces in South Africa witness one of Earth’s largest annual animal migrations, the sardine run. Over the course of this mass migration, hundreds of millions of individuals from the South African population of Pacific sardine, *Sardinops sagax*, form large shoals in the temperate waters of the EC, which then swim north-east along the eastern South African coastline towards the subtropical waters of KZN [2].

Marine species typically migrate to find favourable spawning conditions or to exploit food resources [3,4]. However, the exact dynamic that drives sardine run formation remains unknown. Some studies point to the complex roles that genetic and environmental factors play in forming this mass migration [2,5,6]. Individuals that participate in the sardine run face significant challenges, with the large number of migrating fish attracting a myriad of predators. Furthermore, this event plays a crucial role in the livelihoods and economy of local communities [7].

Recent advances in DNA sequencing technologies and computational resources have made it possible to study the functional content of the genome in a way that was previously impossible. Massively parallel sequencing of the transcriptome from different tissue types has been used to address the biological questions of species from diverse terrestrial and marine environments [8,9,10,11,12,13,14,15].

To better understand the role that genetics plays in the life-history of South African sardines and the formation of the sardine run, extensive genomic resources for this species are needed. One means of doing so is to study the functional content and diversity of the South African sardine genome. Such data not only provide insights into the genetic structure and origin of the individuals that form shoals, but they also shed light on the physiological status of the sardines participating in the migration. 

The functional content of the liver transcriptome in marine vertebrates is particularly informative. Not only do hepatic cells play an essential role in the detoxification of harmful metabolites and anthropogenic pollutants that are accumulating in marine ecosystems at an unprecedented rate, but they are also involved in energy production, energy storage, and in the initiation of various physiological or immune responses necessary to fight pathogens [11,16,17]. 

Migrating animals face a combination of energetic and physiological challenges that differ from those during the non-migrating stages of their life cycles. Several authors have highlighted the critical roles that biochemical pathways in the liver play in migratory species prior to or during migration events [18,19,20]. 

As a first step towards an in-depth understanding of South African sardine biology, we assembled and annotated the first reference transcriptome of liver cells for this species. We further investigated the functional content and genetic diversity among six individuals that participated in the 2019 sardine run.

## 2. Materials and Methods

### 2.1. Animal Ethics Statement

Animal ethics approval for specimen collection was obtained from the Faculty of Science Ethics Committee, University of Johannesburg (approval code:19022015).

### 2.2. Specimen Collection

Six sardines were obtained in KZN during the 2019 sardine run—three individuals from Scottburgh (30°17′12.1″S 30°45′11.5″E) and three from Umgababa (30°08′43.7″S 30°50′00.5″E). All specimens were purchased from artisanal fishers. They had been caught using a beach seine net <15 min prior to specimen collection and had been stored on ice. Approximately 1 mm^3^ of liver tissue was excised immediately upon purchase and was transferred to a 10:1 volume of *RNAlater* solution (Qiagen, Hilden, Germany). The liver samples were then stored at −80 °C until the total RNA was extracted within a week after collection.

### 2.3. Nucleic Acid Extraction, Genomic Library Preparation, and Sequencing

The total RNA was extracted from the liver tissue using a combination of Trizol and Qiagen’s RNeasy Mini Kit. A total amount of 0.4 μg RNA per extraction was used as the template for the RNA library preparation. Complementary DNA (cDNA) libraries were generated using the NEBNext Ultra RNA Library Prep Kit for Illumina (New England Biolabs, MA, USA), following the manufacturer’s recommendations. This was done by first purifying the mRNA from the pool of extracted RNA using poly-T oligo-attached magnetic beads, followed by mRNA fragmentation using divalent cations under an elevated temperature in a NEBNext First Strand Synthesis Reaction Buffer (5×). The first cDNA strand was synthesized using a random hexamer primer and a M-MuLV Reverse Transcriptase (RNase H-) enzyme. Similarly, the synthesis of the second strand of cDNA was performed using DNA Polymerase I and RNase H-. The overhangs were blunt ended by using a combination of exonuclease and polymerase activities. After the adenylation of the 3’ ends of the cDNA fragments, NEBNext Adaptors with a hairpin loop structure were ligated to each library, and the resulting cDNA fragments (~250–300 bp) were purified for an additional step using the AMPure XP system prior to being sequenced on an Illumina HiSeq 4000 platform (San Diego, CA, USA), using a 2 × 150 bp TruSeq PE Cluster Kit v3-cBot-HS following the manufacturer’s instructions.

### 2.4. Transcriptome Assembly, Functional Annotation, and Variant Calling

To construct a comprehensive reference liver transcriptome for South African sardines, the data from the six individuals generated in the current study were combined with sequence data from 20 additional South African sardines’ liver transcriptomes from another study (Teske et al., unpublished. data), prior to quality filtering and transcriptome assembly.

Leading and trailing low-quality sequences, N bases, all base pairs with an average Phred score <25 in a 4-bp sliding window, and Illumina adapter contaminations were removed using Trimmomatic (v0.39) [21]. A reference transcriptome for the liver cells of the South African sardine was assembled de novo using Trinity v2.8.6 [22] by sequentially running the Inchworm, Chrysalis, and Butterfly modules, using their default settings. The most likely reading frame within each transcript was predicted in TransDecoder v5.5.0 [22]. All transcripts were functionally annotated using the Trinotate pipeline [23], with transcripts being searched against known proteins and core metabolic pathways deposited in the Swiss-Prot, NCBI non-redundant protein, and Kyoto Encyclopaedia of Genes and Genomes databases. In addition, and where possible, the protein domains for the assembled transcripts were separately predicted in HMMER v3.1 [24], based on similarity to the deposited protein domains in the Pfam database. Finally, transmembrane proteins and ribosomal RNA were identified using TmHMM v2 [25] and RNAMMER v1.2 [26], respectively.

The gene content of the liver transcriptome was evaluated using BUSCO v4.1.4 and the ray-finned fish core BUSCOs database (actinopterygii_odb10) in transcriptome mode [27].

The quality-filtered sequences of the six sardines collected during the 2019 sardine run were aligned against the assembled reference liver transcriptome using semi-global BWA-MEM aligners [28]. A combination of the SAMtools v1.9 mpileup command [29] and Varscan2 v2.3.7 [30] was used to identify variant sites (Single Nucleotide Polymrphic sites, SNPs). To improve the accuracy of variant calling in Varscan2, the default *p*-value threshold for a variant site was lowered to 0.001.

The functional content and intra-individual genomic variation of the liver transcriptome were investigated by creating two independent datasets. In the first dataset, the abundance for each transcript was estimated using RSEM v1.3.1 [31], and the top 1% of transcripts with the highest normalized level of expression (estimated as the number of transcripts per million mapped reads, TPM) were then selected for more detailed functional analyses. The second dataset consisted of 25% of the assembled transcripts with the highest number of polymorphic sites independent of gene size, as reported in the Varscan2 output files. The enrichment of the generated datasets for particular biological functions in terms of Gene Ontology categories (GO terms) [32] were investigated using the g:Profiler online server [33], with significance thresholds adjusted for multiple testing using g:SCS methods. In the enrichment analysis, the annotated genome of the zebrafish, *Danio rerio* (e101_eg48_p14_baf17f0), was selected as the taxonomically most closely related high-quality annotated gene set against which the statistical significance of the enrichment of the liver transcriptome was tested. Finally, a combination of the WEGO v2.0 online tool [34], REVIGO online server [35], and CirGO [36] were used to visualize the results.

## 3. Results and Discussion

The current study is the first to assemble de novo and annotate a reference transcriptome from the liver tissue of South African sardines.

The Trinity pipeline assembled raw sequences (Supplementary Table S1) into 1,310,530 transcripts with an N50 value of 1578 bp (Supplementary Table S2, Supplementary File sardine.fasta). The gene content analysis of the liver transcriptome identified 73.8% of the complete ray-finned fish BUSCOs within South African sardine liver transcripts. In addition, 8.1% of the predicted BUSCOs were fragmented and 18.1% were missing from the expression profile. Varscan2 identified a total of 68,145 variant sites (SNP density of 0.18 per kb) in the six sardines.

The functional content of the liver transcriptome in South African sardines is consistent with the essential role that hepatic cells play in energy production, energy storage, digestion, secretory, physiological or immune responses to stressors, and the detoxification of harmful chemical compounds [11,16,17].

**Figure 1:**
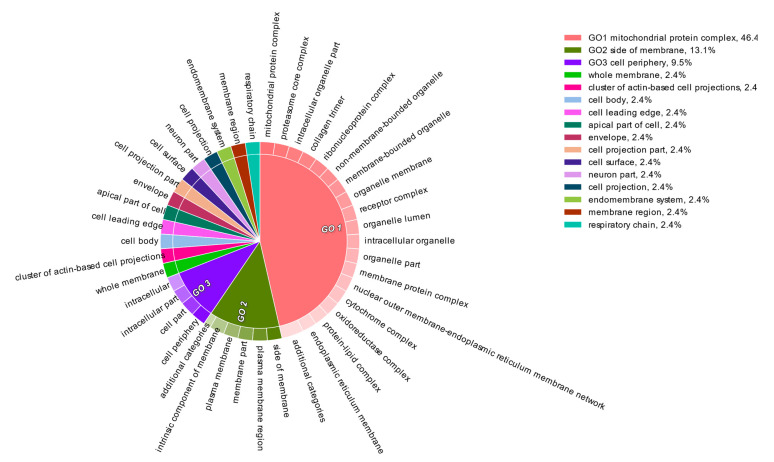
Functional content in terms of the Gene Ontology categorization of “cellular component” for the highly expressed transcripts from the liver transcriptome of South African sardines.

**Figure 2:**
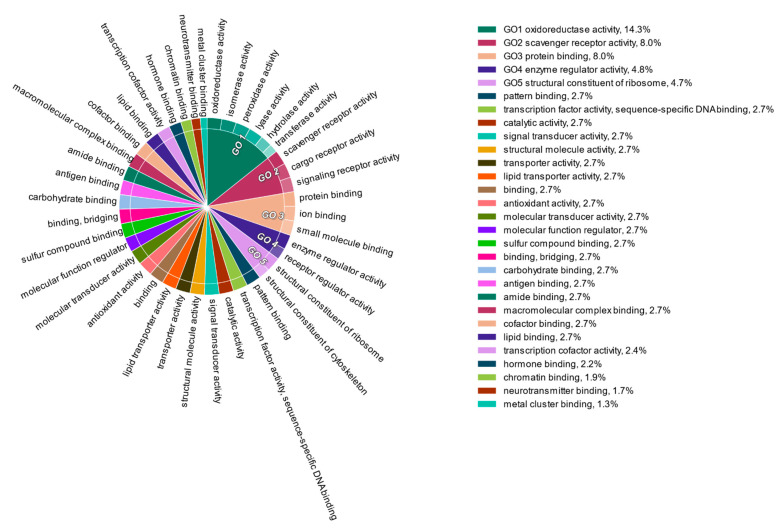
Functional content in terms of the Gene Ontology categorization of “molecular function” for the highly expressed transcripts from the liver transcriptomes of South African sardines.

**Figure 3:**
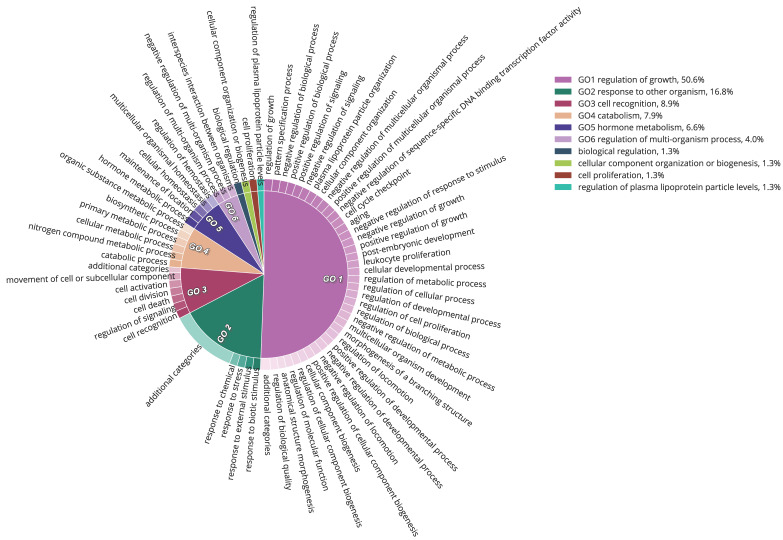
Functional content in terms of the Gene Ontology categorization of “biological process” for the highly expressed transcripts from the liver transcriptome of South African sardines.

The liver transcripts were heavily enriched for molecular functions such as binding, catalytic, regulatory, and transducer activity. Similarly, biological processes such as those involved in the cellular macromolecule metabolic processes, physiological responses to internal and external stimuli, immune responses, cell communication, and cell signaling were significantly overrepresented (Figure 1, Figure 2 and Figure 3).

The KEGG pathway analysis of the South African sardine liver transcriptome highlighted that biochemical pathways that function as signal transducers (*n* = 3812), those involved in general metabolic pathways (*n* = 3579), in the immune system (*n* = 1475), and in the endocrine system (*n* = 1473) are active in the liver cells. Not surprisingly, a large number of metabolic pathways that are associated with the biodegradation of xenobiotic substances (*n* = 210), amino acid or lipid metabolism (*n* = 1134), the digestive system (*n* = 442), excretory system (*n* = 121), and environmental adaptation (*n* = 289) were also identified (Table 1).

In the dataset that consists of the 1% highly expressed genes in the South African sardine liver transcriptome (Supplementary Table S3), genes such as glutathione S-transferase (GTS) are a well-studied family of liver enzymes that play a critical role in the detoxification of electrophilic toxins [37]. High expression levels for these genes have been reported from both healthy liver cells and hepatic cells after exposure to toxic substances in different fish species, such as plaice (*Pleuronectes platessa*), flounder (*Platichthys flesus*), big-belly seahorse (*Hippocampus abdominalis*), and grass carp (*Ctenopharyngodon idella*) [38,39,40,41,42,43,44].

Similarly, a high expression of zona pellucida glycoprotein 3 (*zp3*) gene, which is involved is sperm–oocyte binding, was also reported from the liver cells of trout (*Oncorhynchus mykiss*), salmon (*Salmo salar*), and winter flounder (*Pseudo-pleuronectes americanus*), as well as from the ovaries of zebrafish and common carp (*Cyprinus carpio*) [45,46]. Conner and Hughes [46] suggested that an ancient gene duplication event in teleost evolution resulted in two copies of these genes being independently active in ovaries and liver tissues, and that the expression level in each organ depends on a complex regulatory mechanism that involves cross-talks between tissue types and tissue-specific regulatory mechanisms. Among other highly expressed genes, the pleiotropic gene, X-box binding protein 1 (*XBP1*), triggers an endoplasmic stress response in zebrafish [47], and protein *SelK* (selenoprotein K) initiates innate immunological responses in common carp and rainbow trout (*Oncorhynchus mykiss*) [48]. The expression levels of *cirbp* (cold inducible RNA binding protein) and *csde1* (cold shock domain containing E1 genes) in the sardines’ livers are noteworthy, as both of these genes are involved in physiological responses to cold temperature. In the zebrafish, *cirbp* acts as a molecular chaperone that aids in unfolding RNA secondary structures, especially in colder water [49]. In other vertebrates, the upregulation of the *csde1* gene has been reported in response to the stress experienced during rapid temperature decrease [50] through complex nucleic acid-binding activities [51].

A number of genes that are involved in oxygen transport (such as *Hbb*) and energy production pathways in the mitochondrion (such as *ATP5H*, *ATP5I*, *ATP5J*, *ATP5L*, *CYB5*, *CYC*, *HOGA1*, *MSRB2*, *UCP1*, *UCP2*, and *UCP3*) demonstrated comparatively high expression levels in the South African sardine liver transcriptome. While high expressions of the genes that are involved in energy production and those that modulate the physiological or immunological responses to the environmental stressors in the hepatic cells is not surprising, the extent to which the observed pattern may provide migrating fish with the sudden energetic and physiological requirements prior to or during migration events is yet to be fully understood, and this requires more detailed comparative studies between sardines that participate in the sardine run and those that do not. Sharma et al. [18] described enrichment in similar metabolic pathways within the liver transcriptome of migratory birds participating in seasonal migrations.

Among the highly polymorphic genes in the South African sardines’ liver transcriptome (Supplementary Table S4), the product of the arsenic (+3 oxidation state) methyltransferase gene (*AS3MT*) metabolizes naturally-occurring or xenobiotic arsenic compounds into the easily-excretable monomethylarsonic and dimethylarsonic products [52]. Similarly, glutathione S-transferase pi 1 (*GSTP1*) and glutathione S-transferase pi 2 (*GSTP2*) genes catalyse glutathione into electrophilic compounds, which are essential in the detoxification process [53]. Polymorphism in *GREB1* is notable, as the expression of this gene has been shown to synchronize the arrival of sexually mature or premature salmonid fish to their spawning grounds [54]. Other highly polymorphic genes are involved in a wide range of metabolic, regulatory, and catalytic activities.

We suggest that many of the genes predicted from South African sardines’ liver transcriptome perform similar functions as they do in other vertebrate species. Our results also indicate that the high expression level and polymorphism in the glutathione S-transferase (*GTS*) gene can make their products a potential biomarker that is both easy to detect and variable enough to reflect the health status of different individuals. However, more physiological and toxicological studies are needed in order to evaluate sensitivity and specificity of this gene as a suitable biomarker, similar to how it has been used as a biomarker in other species [55,56].

**Figure 4:**
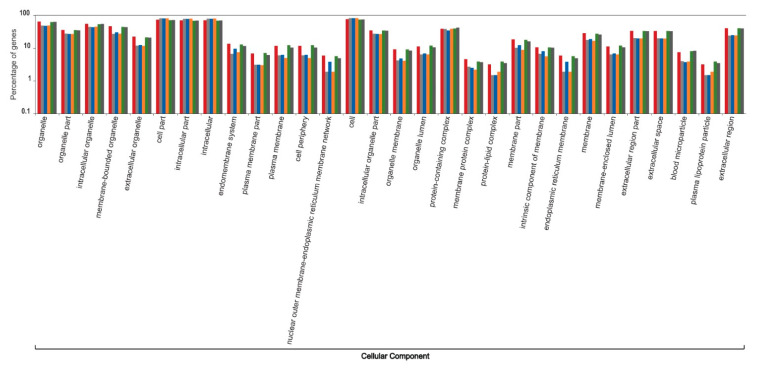
Intra-individual variation in the functional content of the top one percent of highly expressed transcripts within the liver transcriptome of South African sardines in terms of the Gene Ontology categorization “cellular component”, shown for six individuals that participated in the 2019 sardine run. Each individual is represented by a unique colour. Values on the Y axis are log transformed.

**Figure 5:**
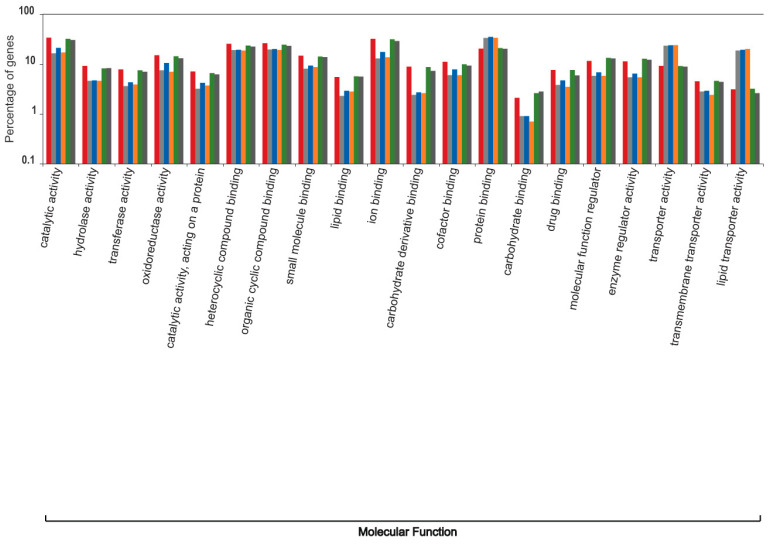
Intra-individual variation in the functional content of the top one percent of highly expressed transcripts within the liver transcriptome of South African sardines in terms of the Gene Ontology categorization “molecular function”, shown for six individuals that participated in the 2019 sardine run. Each individual is represented by a unique colour. Values on the Y axis are log transformed.

**Figure 6:**
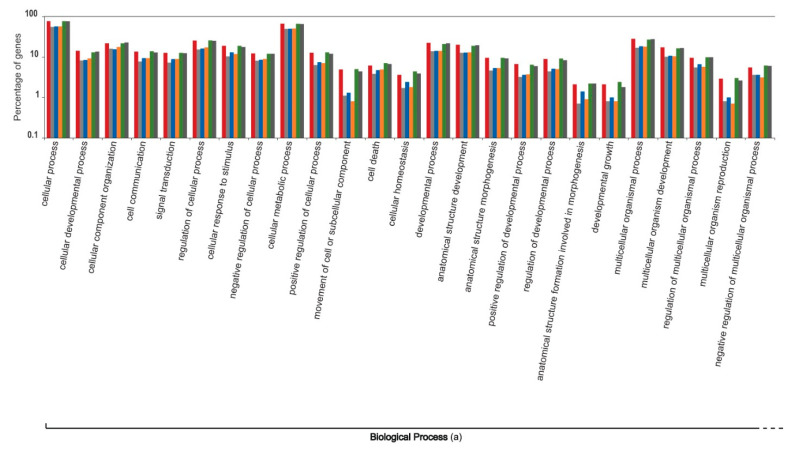
Intra-individual variation in the functional content of the top one percent of highly expressed transcripts within the liver transcriptome of South African sardines in terms of the Gene Ontology categorization “biological process”, shown for six individuals that participated in the 2019 sardine run. Each individual is represented by a unique colour. Values on the Y axis are log transformed.

**Figure 7:**
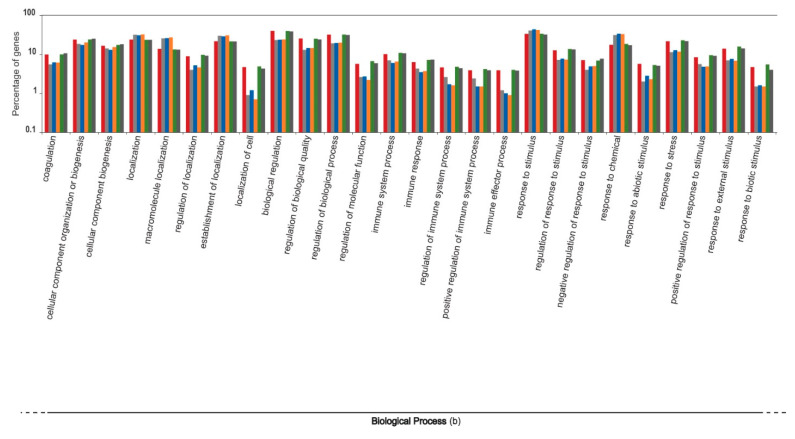
Intra-individual variation in the functional content of the top one percent of highly expressed transcripts within the liver transcriptome of South African sardines in terms of the Gene Ontology categorization “biological process”, shown for six individuals that participated in the 2019 sardine run. Each individual is represented by a unique colour. Values on the Y axis are log transformed (continued from Figure 6).

**Figure 8:**
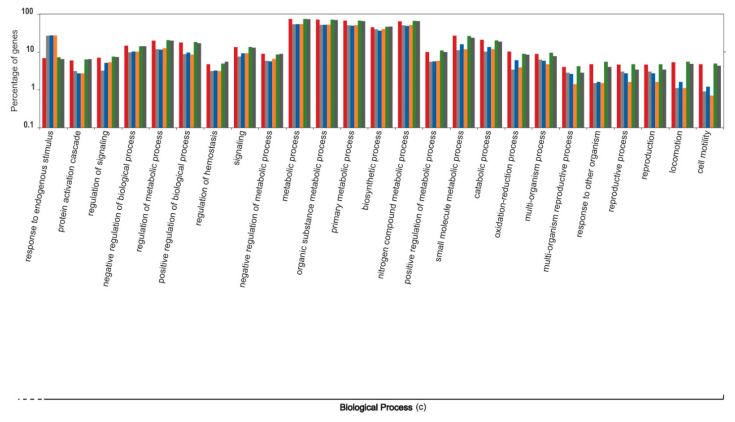
Intra-individual variation in the functional content of the top one percent of highly expressed transcripts within the liver transcriptome of South African sardines in terms of the Gene Ontology categorizations “biological process”, shown for six individuals that participated in the 2019 sardine run. Each individual is represented by a unique colour. Values on the Y axis are log transformed (continued from Figure 7).

The functional content of highly expressed transcripts from the six individuals that participated in the sardine run showed a heterogeneous variation (Figure 4, Figure 5, Figure 6, Figure 7 and Figure 8). The molecular component category showed minimal variation among six individuals. However, the variations in the two other gene ontology categories, molecular function and biological process, were comparatively more pronounced. Molecular functions, such as lipid transporter and binding activity, carbohydrate and carbohydrate derivative binding activity, and a series of biochemical pathways that are involved in various catalytic activities acting on the proteins and on other biomolecules, showed comparatively high intra-individual variations. Similarly, biological processes such as those that modulate the physiological and transcriptomic responses to biotic stimuli, immune effector processes, multicellular organism reproduction, developmental growth, cell motility, locomotion, and responses to other organisms varied considerably among individuals. The significance of the observed pattern in the functional content of the liver transcriptome among individual sardines, which were subjected to the same environmental stimuli and stressors during migration and capture, requires more detailed study of sardine populations under controlled conditions, which lies beyond the scope of the current study. 

Overexploitation of marine species, human-mediated climate change, and pollution threaten marine biodiversity globally. Marine species face an uncertain future, and their survival depends on their ability to adapt to this rapidly evolving environment. Many migratory species have already changed the route and timing of their migrations as a result of anthropogenic changes in their environment [57,58,59]. The long-term negative effects of human activities on the sardine run are yet to be fully understood. Mhlongo et al. [60] showed that an increase in sea surface temperature can negatively influence the spawning behaviour of sardines, which typically spawn at water temperatures between 16.0 °C and 22.0 °C. Other studies predict that the current rate of increase in sea surface temperature in EC and KZN could result in massive breeding failure, starvation, and, eventually, the complete disappearance of the sardine run [61,62].

Evaluating the functional content of an organism’s genome provides us with an in-depth understanding of the complex dynamics of local adaptations, which form an integral part of species’ adaptive responses to challenges that they will face in the near future. The present study provides a starting point for understanding the adaptive potential of South Africa’s sardine population to global change.

## Figures and Tables

**Table 1:** Description and number of occurrences of Kyoto Encyclopaedia of Genes and Genomes (KEGG) metabolic pathways predicted from the liver transcriptome of South African sardine.

Name of the Pathway	Occurrences
Signal transduction	3812
Global and overview maps	3579
Immune system	1475
Endocrine system	1473
Transport and catabolism	1424
Carbohydrate metabolism	774
Nervous system	750
Amino acid metabolism	647
Lipid metabolism	487
Cellular community—eukaryotes	468
Folding, sorting, and degradation	448
Digestive system	442
Translation	416
Energy metabolism	383
Glycan biosynthesis and metabolism	376
Metabolism of cofactors and vitamins	351
Replication and repair	299
Environmental adaptation	289
Development and regeneration	284
Circulatory system	249
Nucleotide metabolism	226
Xenobiotics biodegradation and metabolism	210
Cell motility	210
Transcription	181
Sensory system	164
Ageing	150
Metabolism of other amino acids	148
Biosynthesis of other secondary metabolites	125
Excretory system	121
Metabolism of terpenoids and polyketides	86

## Data Availability

Raw sequences for this study have been submitted to the NCBI Short Reads Archives (SRA) under Bio Project ID PRJNA701779. The assembled hepatic transcriptome is available in the Supplementary File sardine.fasta.

## Supplementary Materials

The following are available online at https://www.mdpi.com/2073-4425/12/3/368/s1: Table S1: the number of quality-filtered sequences used for the liver assembly; Table S2: summary statistics of the liver transcriptome assembly; Table S3: gene symbols and full names of 1% highly expressed transcripts predicted from the liver transcriptome; Table S4: gene symbols and the full name of highly polymorphic transcripts (independent of their size) from the liver transcriptome.
