# Supplementary material for: Transcriptomic Diversity in the Livers of South African Sardines Participating in the Annual Sardine Run
Source: Genes (Basel). 2021 Mar 4;12(3):368. doi: 10.3390/genes12030368 (PMC8001748; doi:10.3390/genes12030368)
Supplement: Supplementary file 1 [file genes-12-00368-s001.zip › genes-1100101-supplementary-final.docx]

**Table S1.** Number of quality-filtered reads that were used for the liver transcriptome of the South African sardine.

| **Scheme 001009190.** | **# of Reads** |
| --- | --- |
| C001009190* | 16623287 |
| C001020202* | 14993874 |
| C001033216* | 33980014 |
| C0064831* | 5404146 |
| C0064841* | 26912580 |
| C00806160* | 23310210 |
| C00806164* | 14510495 |
| C00806165_S5* | 14402814 |
| C00806168_S6* | 50547420 |
| C0085906* | 15255885 |
| C00859-1* | 52726026 |
| C00859-2* | 52325327 |
| C00859-3_2* | 43715023 |
| C00859-4* | 53295469 |
| C00859-5* | 49157512 |
| C0091368* | 12254936 |
| C00931102_S3* | 17683932 |
| C00971121* | 16560234 |
| C00981145_S4* | 14575022 |
| C00991168* | 13755849 |
| SasaSCB2_1** | 51987559 |
| SasaSCB4** | 50303751 |
| SasaSCB8** | 48205569 |
| SasaUGB1** | 51386791 |
| SasaUGB2_1** | 51688103 |
| SasaUGB5** | 50242076 |

*Quality-filtered sequences from Teske et al., ** Raw data generated for this study.

**Table S2.** Summary statistics of the South African sardine (*Sardinops sagax*) liver transcriptome assembly.

| **Statistics** | **Number** |
| --- | --- |
| # contigs (≥ 0 bp) | 1310530 |
| # contigs (≥ 1000 bp) | 127680 |
| # contigs (≥ 5000 bp) | 4009 |
| # contigs (≥ 10000 bp) | 260 |
| # contigs (≥ 25000 bp) | 5 |
| # contigs (>= 50000 bp) | 1 |
| Total length (≥ 0 bp) | 667660994 |
| Total length (≥ 1000 bp) | 262353082 |
| Total length (≥ 5000 bp) | 27004107 |
| Total length (≥ 10000 bp) | 3378200 |
| Total length (≥ 25000 bp) | 174028 |
| Total length (≥ 50000 bp) | 53575 |
| # contigs | 295197 |
| Largest contig | 53575 |
| Total length | 376981267 |
| GC (%) | 46.89 |
| N50 | 1578 |
| N75 | 876 |
| L50 | 68500 |
| L75 | 149493 |
| # N's per 100 kbp | 0.00 |

**Table S3.** Uniport symbol and full names of the 1% of the highly expressed transcripts predicted from South African sardine (*Sardinops sagax*) liver transcriptome.

| **Gene Symbol** | **Full Name** |
| --- | --- |
| *A1CF* | Apobec1 complementation factor |
| *ACADM* | acyl-Coenzyme A dehydrogenase |
| *ACTB* | Actin, cytoplasmic 1 |
| *AFP4* | Type IV antifreeze protein A |
| *AIMP2* | Aminoacyl tRNA synthetase complex-interacting multifunctional protein 2 |
| *ALDH2* | Aldehyde dehydrogenase 2 |
| *ALDOB* | Aldolase b, fructose-bisphosphate |
| *ALLC* | Allantoicase |
| *AMBP* | Alpha-1-microglobulin/bikunin precursor |
| *ANXA4* | Annexin A4 |
| *APOA* | Apolipoprotein A-I |
| *APOA1* | Apolipoprotein A-I |
| *APOA4* | Apolipoprotein A-IV b, tandem duplicate 1; Apolipoprotein A-IV |
| *APOB* | Apolipoprotein Ba |
| *APOC1* | Apolipoprotein C-I like |
| *APOC2* | Apolipoprotein C-II |
| *APOEB* | Apolipoprotein Eb |
| *AQP12* | Aquaporin |
| *AQP9* | Aquaporin 9b |
| *ATF4* | Cyclic AMP-dependent transcription factor ATF-4 |
| *ATG12* | Ubiquitin-like protein ATG12 |
| *ATP5H* | ATP synthase subunit d, mitochondrial |
| *ATP5I* | ATP synthase, H+ transporting, mitochondrial F0 complex, subunit e, duplicate b |
| *ATP5J* | ATP synthase-coupling factor 6, mitochondrial |
| *ATP5L* | ATP synthase, H+ transporting, mitochondrial F0 complex, subunit g |
| *ATP6* | ATP synthase subunit a |
| *BAF* | Barrier-to-autointegration factor |
| *BNIP3* | BCL2/adenovirus E1B interacting protein 3 |
| *BTF3* | Basic transcription factor 3 |
| *C1QL2* | Complement component 1, q subcomponent-like 2 |
| *C1R* | Complement component 1, r subcomponent |
| *C1S* | Complement component 1, s subcomponent |
| *CALU* | Calumenin-B |
| *CATD* | Ctsd protein |
| *CBLN2* | Cerebellin 2a precursor |
| *CBS* | Cystathionine-beta-synthase b |
| *CCNB1* | Cyclin B1 |
| *CCNG1* | Cyclin G1 |
| *CCNI* | Cyclin I |
| *CDK12* | Cyclin-dependent kinase 12 |
| *CHAC1* | Glutathione-specific gamma-glutamylcyclotransferase 1 |
| *CIDEB* | Cell death-inducing DFFA-like effector b |
| *CIRBP* | Cold inducible RNA binding protein |
| *CISD1* | CDGSH iron sulfur domain 1 |
| *CNBP* | CCHC-type zinc finger, nucleic acid binding protein a |
| *CNTFR* | Ciliary neurotrophic factor receptor |
| *COX1* | Cytochrome c oxidase subunit 1 |
| *COX2* | Cytochrome c oxidase subunit 2 |
| *COX3* | Cytochrome c oxidase subunit 3 |
| *COX5A* | Cytochrome c oxidase subunit Vaa |
| *CRIM1* | Cysteine-rich motor neuron 1 protein |
| *CSDE1* | Cold shock domain containing E1, RNA-binding |
| *CYB5* | Cytochrome b5 type A (microsomal) |
| *DDX21* | DEAD (Asp-Glu-Ala-Asp) box polypeptide 21 |
| *DGAT2* | Diacylglycerol O-acyltransferase 2 |
| *DPYD* | Dihydropyrimidine dehydrogenase [NADP(+)] |
| *E2F3* | E2F transcription factor 3 |
| *EBP* | Emopamil binding protein (sterol isomerase) |
| *EDF1* | Endothelial differentiation-related factor 1 homolog |
| *EDIL3* | EGF-like repeats and discoidin I-like domains 3 |
| *EF1A* | Elongation factor 1-alpha |
| *EIF1B* | Eukaryotic translation initiation factor 1B |
| *EIF3B* | Eukaryotic translation initiation factor 3 subunit B |
| *EIF3C* | Eukaryotic translation initiation factor 3 subunit C |
| *EIF3D* | Eukaryotic translation initiation factor 3 subunit D |
| *EIF3F* | Eukaryotic translation initiation factor 3, subunit F |
| *EIF3G* | Eukaryotic translation initiation factor 3 subunit G |
| *EIF3H* | Eukaryotic translation initiation factor 3 subunit H-A |
| *EIF3I* | Eukaryotic translation initiation factor 3 subunit I |
| *EIF3M* | Eukaryotic translation initiation factor 3 subunit M |
| *EPD* | Ependymin |
| *ERBB2* | Receptor protein-tyrosine kinase |
| *ETFA* | Electron-transfer-flavoprotein, alpha polypeptide |
| *ETFB* | Electron-transfer-flavoprotein, beta polypeptide |
| *F13B* | Coagulation factor XIII, B polypeptide |
| *FABP4* | Fatty acid binding protein 11a |
| *FADS2* | Fatty acid desaturase 2 |
| *FAT4* | FAT atypical cadherin 4 |
| *FETUA* | alpha-2-HS-glycoprotein |
| *FETUB* | Fetuin B |
| *FKBP2* | Peptidylprolyl isomerase |
| *FOLR1* | Zgc:165502; Folate receptor 1 (adult) |
| *FOS* | V-fos FBJ murine osteosarcoma viral oncogene homolog |
| *FTCD* | Formiminotransferase cyclodeaminase |
| *FZD1* | Frizzled homolog 1 |
| *G6PC* | Glucose-6-phosphatase a, catalytic, tandem duplicate 1 |
| *GCDH* | glutaryl-Coenzyme A dehydrogenase |
| *GGCT* | Gamma-glutamyl cyclotransferase b |
| *GPX3* | Glutathione peroxidase 3 (plasma) |
| *GPX4* | Glutathione peroxidase 4a |
| *GRHPR* | Novel protein similar to human glyoxylate reductase/hydroxypyruvate reductase (GRHPR) |
| *GRN* | Apelin receptor B |
| *GSTP1* | Glutathione S-transferase pi 1 |
| *GSTP2* | Glutathione S-transferase pi 2 |
| *HABP2* | Hyaluronan binding protein 2 |
| *HBA* | Hemoglobin alpha embryonic-1 |
| *HBB* | Hemoglobin subunit beta-1 |
| *HEBP2* | Heme binding protein 2 |
| *HGD* | Homogentisate 1,2-dioxygenase |
| *HGFL* | Phosphoinositide-3-kinase-interacting protein 1 |
| *HINT1* | Histidine triad nucleotide binding protein 1 |
| *HMCN1* | Hemicentin 1 |
| *HMGB2* | High-mobility group box 2a |
| *HOGA1* | 4-hydroxy-2-oxoglutarate aldolase, mitochondrial |
| *HSP70* | Heat shock cognate 70-kd protein, tandem duplicate 3 |
| *IDI1* | Isopentenyl-diphosphate delta isomerase 1 |
| *IGF1A* | Insulin-like growth factor 1 |
| *IGF2* | Insulin-like growth factor 2a |
| *ISOC2* | Isochorismatase domain containing 2 |
| *ITIH2* | Inter-alpha-trypsin inhibitor heavy chain 2 |
| *ITIH4* | Inter-alpha-trypsin inhibitor heavy chain family, member 4 |
| *ITM2B* | Integral membrane protein 2Ba |
| *JUN* | Jun proto-oncogene, AP-1 transcription factor subunit; Jun proto-oncogene |
| *KMO* | Kynurenine 3-monooxygenase |
| *LDLR* | Low density lipoprotein receptor |
| *LECT2* | Leukocyte cell-derived chemotaxin 2 like |
| *LRP2* | Low density lipoprotein receptor-related protein 2a |
| *LSM6* | LSM6 homolog, U6 small nuclear RNA associated |
| *MASP1* | Mannan-binding lectin serine peptidase 1 |
| *MBL2* | Hexose-binding lectin 3 |
| *MCL1* | MCL1, BCL2 family apoptosis regulator a |
| *MFAP4* | Microfibrillar-associated protein 4 |
| *MIOX* | Myo-inositol oxygenase |
| *MMP2* | Matrix metalloproteinase 2 |
| *MSMO1* | Methylsterol monooxygenase 1 |
| *MSRB2* | Methionine-R-sulfoxide reductase B2, mitochondrial |
| *MT* | Metallothionein-2 |
| *MYL9* | Myosin, light chain 9a, regulatory |
| *NACA* | Nascent polypeptide-associated complex subunit alpha |
| *NDR1A* | Neurogenic differentiation factor 6-A |
| *NDRG1* | N-myc downstream regulated gene 1a |
| *NDRG3* | N-myc downstream regulated family member 3a |
| *NLRP1* | NLR family, pyrin domain containing 1 |
| *NSA2* | NSA2 ribosome biogenesis homolog |
| *NUPR1* | Nuclear protein 1 |
| *OAT* | Solute carrier family 22 member 6 |
| *OAZ1* | Ornithine decarboxylase antizyme 1 |
| *OVOS* | Ovo-like zinc finger 3 |
| *PBLD* | Phenazine biosynthesis-like protein domain containing |
| *PBLD2* | Phenazine biosynthesis-like protein domain containing |
| *PCSK5* | Proprotein convertase subtilisin/kexin type 5b |
| *PCSK6* | Proprotein convertase subtilisin/kexin type 6 |
| *PDCL3* | Phosducin-like 3 |
| *PDIA3* | Protein disulfide isomerase family A, member 3 |
| *PDK1* | Pyruvate dehydrogenase kinase, isozyme 1 |
| *PDK2* | Pyruvate dehydrogenase kinase, isoenzyme 2 |
| *PDK3* | Pyruvate dehydrogenase kinase, isozyme 3a |
| *PDK4* | Pyruvate dehydrogenase kinase, isozyme 4 |
| *PGK1* | Phosphoglycerate kinase 1 |
| *PGM1* | Phosphoglucomutase 1 |
| *PIGR* | Polymeric immunoglobulin receptor |
| *PLIN2* | Perilipin 2; Belongs to the perilipin family |
| *PNKP* | Polynucleotide kinase 3'-phosphatase |
| *PNO1* | RNA-binding protein PNO1 |
| *POLG* | Polymerase (DNA directed), gamma |
| *POLN* | Polymerase (DNA directed) nu; HAUS augmin-like complex, subunit 3 |
| *PPIA* | Peptidylprolyl isomerase A (cyclophilin A) |
| *PPIB* | Peptidyl-prolyl cis-trans isomerase |
| *PPIH* | Peptidyl-prolyl cis-trans isomerase |
| *PRDX1* | Peroxiredoxin 1 |
| *PRDX6* | Peroxiredoxin 6 |
| *PRG4* | Proteoglycan 4b |
| *PRKRA* | Interferon-inducible double-stranded RNA-dependent protein kinase activator A homolog |
| *PROC* | Protein C (inactivator of coagulation factors Va and VIIIa), a |
| *PROP* | PROP paired-like homeobox 1 |
| *PTGDS* | Lipocalin-type prostaglandin D synthase-like protein |
| *PTPRD* | Uncharacterized protein |
| *RCL1* | RNA terminal phosphate cyclase-like 1 |
| *RDH12* | Retinol dehydrogenase 12 (all-trans and 9-cis) |
| *RDH14* | Retinol dehydrogenase 14b (all-trans/9-cis/11-cis) |
| *RDHE2* | Short chain dehydrogenase/reductase family 16C, member 5 |
| *RGL1* | Ral guanine nucleotide dissociation stimulator-like 1 |
| *RGL2* | Ral guanine nucleotide dissociation stimulator-like 2 |
| *ROMO1* | Reactive oxygen species modulator 1 |
| *RTN1* | Reticulon 1b |
| *RTN1A* | Reticulon 1a |
| *RTN4* | Reticulon 4a |
| *SAT1* | Spermidine/spermine N1-acetyl transferase 1b |
| *SC5D* | sterol-C5-desaturase |
| *SDC2* | Syndecan |
| *SELK* | Selenoprotein K |
| *15-Sep* | Selenoprotein F |
| *SERP2* | Stress-associated endoplasmic reticulum protein |
| *SHBG* | Sex hormone binding globulin |
| *SPP24* | Secreted phosphoprotein 2, 24kDa |
| *SRPK3* | SRSF protein kinase 3 |
| *TCTP* | Translationally-controlled tumor protein homolog |
| *THRB* | Thyroid hormone receptor beta |
| *TOB1* | Transducer of ERBB2, 1b |
| *TTC36* | Tetratricopeptide repeat domain 36 |
| *UBB* | Ubiquitin C |
| *UCP1* | Uncoupling protein 1 |
| *UCP2* | Mitochondrial uncoupling protein 2 |
| *UCP3* | Uncoupling protein 3 |
| *UPP1* | Uridine phosphorylase |
| *UPP2* | Uridine phosphorylase |
| *URAD* | 2-oxo-4-hydroxy-4-carboxy-5-ureidoimidazoline decarboxylase |
| *USMG5* | Up-regulated during skeletal muscle growth 5 homolog |
| *UTP18* | UTP18 small subunit (SSU) processome component |
| *VTCN1* | V-set domain containing T cell activation inhibitor 1 |
| *XBP1* | X-box binding protein 1 |
| *XDH* | Xanthine dehydrogenase |
| *ZP3* | Zona pellucida glycoprotein 3, tandem duplicate 2 |

**Table 4.** Uniport symbol and full names of 25% highly polymorphic transcripts predicted from South African sardine *(Sardinops sagax*) liver transcriptome. Only transcripts that were annotated to orthologs in the zebra fish (*Danio rerio*) genome were shown.

| **QueryItem** | **Annotation** |
| --- | --- |
| AS3MT | Arsenic (+3 oxidation state) methyltransferase |
| CGNL1 | Cingulin-like 1 |
| GREB1 | Growth regulation by estrogen in breast cancer 1 |
| GSTP1 | Glutathione S-transferase pi 1 |
| GSTP2 | Glutathione S-transferase pi 2 |
| HGFL | Phosphoinositide-3-kinase-interacting protein 1 |
| LARP1 | La ribonucleoprotein domain family, member 1 |
| LST2 | Lateral signaling target protein 2 homolog |
| MPRIP | Myosin phosphatase Rho interacting protein |
| PCSK6 | Proprotein convertase subtilisin/kexin type 6 |
| RHOF | Ras homolog gene family, member F |
| SELPB | Selenoprotein Pb |
| SPF30 | Survival of motor neuron-related-splicing factor 30 |
| VIP2 | Vasoactive intestinal peptide |
